# The Bauhaus: its influence on hospital design and wine labels

**DOI:** 10.1038/s41409-019-0589-y

**Published:** 2019-06-12

**Authors:** Shaun R. McCann

**Affiliations:** Haematology Emeritus, Trinity College, University of Dublin, Dublin, Ireland

‘Less is more’

Ludwig Mies van der Rohe. German – American architect and one of the pioneers of Modernist architecture (1886–1969)

This year marks the hundredth Anniversary of the founding of the ‘Bauhaus’ by German architect Walter Gropius and as Rebecca Watson writing in the weekend FT 6/7th April 2019 says: ‘The Bauhaus was exceptional: it never really went away [1]. She goes on to say: ‘The Bauhaus endures because its principles can be boiled down to universal ideas: functionality, simplicity and innovation’. The movement only lasted 14 years but its legacy lives on. The movement fell foul of the Nazis and many of those associated with it emigrated to the United States, the Palestine Mandate (now Israel) and South Africa. Modern day Tel Aviv contains numerous ‘Bauhaus’ buildings and is a UNESCO heritage site. Although the original Bauhaus movement had socialist leanings, much of this philosophical view was abandoned after 1936. Apart from its obvious influence on architecture (e.g. the Seagram building and the MetLife buildings on Park Avenue) today many of the designs are incorporated into artefacts/furniture to be found in places like the MoMA (Museum of Modern Art) gift shop in New York. In the UK Conran’s Habitat shops and more latterly IKEA probably represent ‘modern design’, which is widely accessible. The spectrum of influence includes the ubiquitous iPhone and many items of table ware.

Did the ‘Bauhaus’ movement influence hospital design? Yes. Tony Monk quotes ‘Studies in the Function and Design of Hospitals’ associated with Bristol University in his book ‘Hospital Builders’ [2] which claims that; ‘it (hospital design) was based on the then Modern Movement principles that form follows function’. The most famous ‘Bauhaus’ hospital design, but never realised, was Le Corbusier’s Venice Hospital project. Many hospitals in the UK and Ireland were in Victorian buildings and in Ireland some were even housed in eighteenth century houses. The Modernist movement concentrated on functionality; however, this was an aspect of hospitals not widely considered prior to 1900. In Siena, Santa Maria della Scala, one of the oldest hospitals in Europe, for example, was in use as a hospital until 1996.

**Fig. 1:**
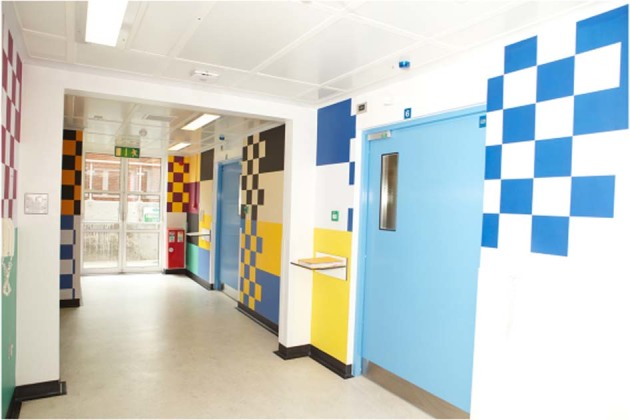
Painted walls of transplant unit St James’ Hospital. Courtesy Anthony Edwards, Senior Clinical Photographer

Like many things from music to furniture not everybody approved the Modernist movement. Some believed that hospital design had become ‘too clinical’ and lacked ‘humanity’. Dr Diana Anderson, an architect and physician, has been in the forefront in trying to increase awareness of the importance of including staff and patients’ wellbeing into hospital design [3]. I fully agree with Dr Anderson and tried to enhance the environment in The National Bone Marrow Transplant Unit in St James’ Hospital some years ago. I am not hubristic enough to equate myself with the great figures of The Bauhaus but in 2005 I introduced a multimedia intervention in the National Bone Marrow Transplant Unit in St James’ Hospital, Dublin. It was an attempt to make the environment less ‘clinical’ for patients and staff. I engaged an artist to paint the corridors and air-lock (Fig. 1). The artist offered to paint the walls in the colours of county football teams. He reasoned that everybody (even nuns) would identify with their own county colours. He was correct and this encouraged me to be braver. I then formed a team, which came up with the idea of creating a virtual window, which consisted of a variety of still and moving images in patients’ rooms. A randomised prospective psychological study was conducted with financial support from the Irish Cancer Society and The Bone Marrow for Leukaemia Trust. The study and its results were published in 2011 and showed a beneficial effect of ‘Open Window’ on anxiety and depression levels but most importantly on levels of expectation among patients on the experience of having a bone marrow transplant [4].

**Fig. 2:**
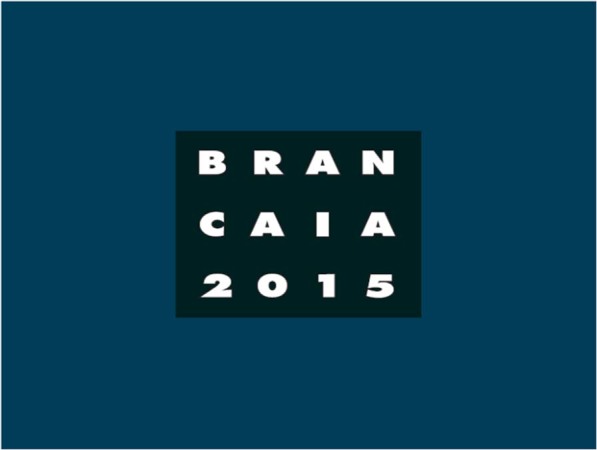
Wine label from La Brancaia, Tuscany

**Fig. 3:**
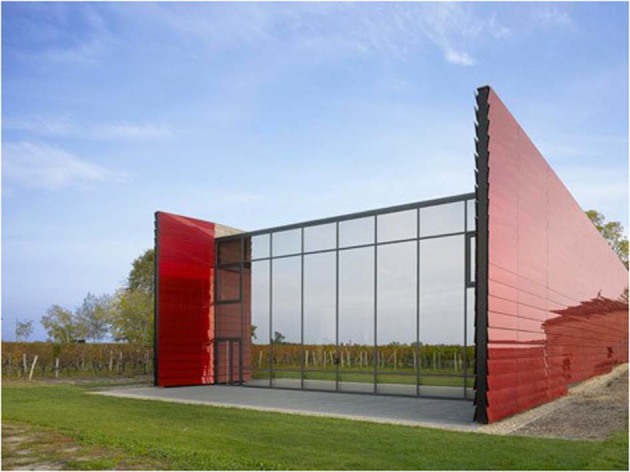
Photograph 3 A Cantina Château La Dominique by Jean Nouvel

**Fig. 4:**
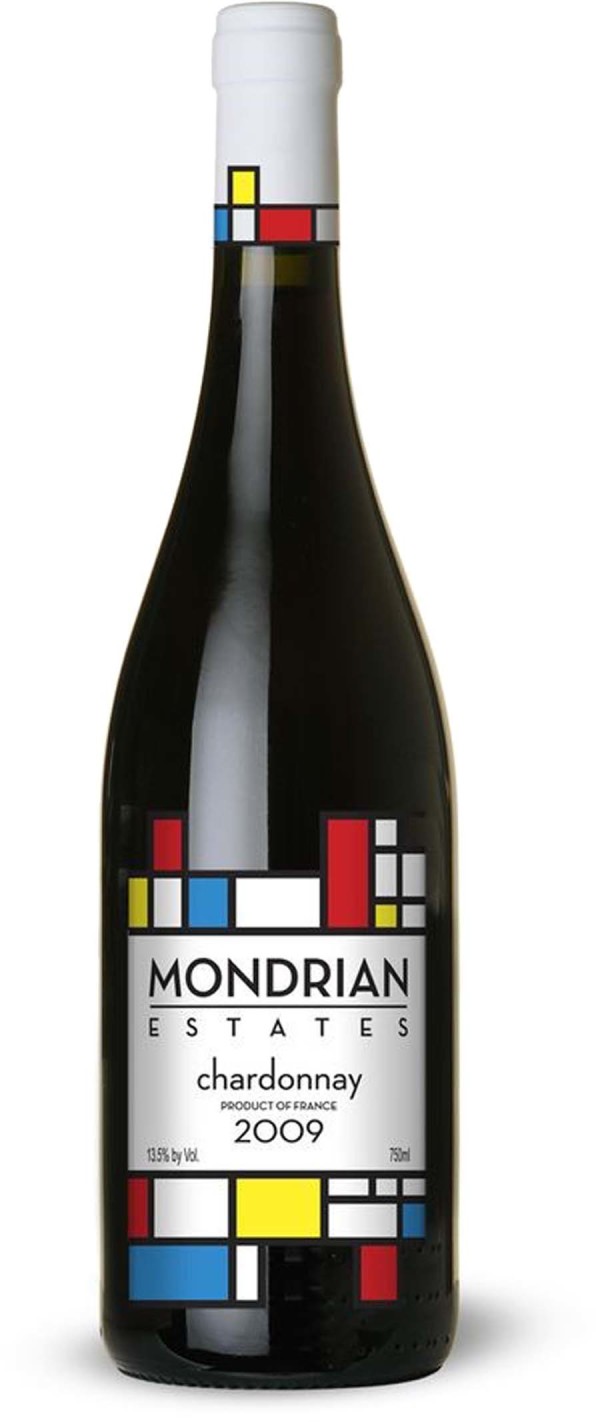
Château la Dominique, Chardonnay

What about wine glasses and wine labels and the Bauhaus? The Riedel company, one of the foremost makers of wine glasses states that their designs have been influenced by the ‘Bauhaus’. Wine labels are highly polymorphic and many producers still favour castles and coats of arms. However, some labels are influenced by Bauhaus design. A recent conversation with Bruno Widmer from La Brancaia in Tuscany confirmed this (Fig. 2). ‘*We don’t live in a castle in Tuscany and we have no coat of arms so with my designer we produced a label which is still in use 30 years later*’ remarks by Lucia, sales and export manager at La Brancaia, attributed to owner Bruno Widmer. The ‘cantina’, designed by Jean Nouvel, and wine labels from Château la Dominique in Saint—Émilion have clearly been influenced (Figs. 3 and 4) by the Bauhaus.

**Fig. 5:**
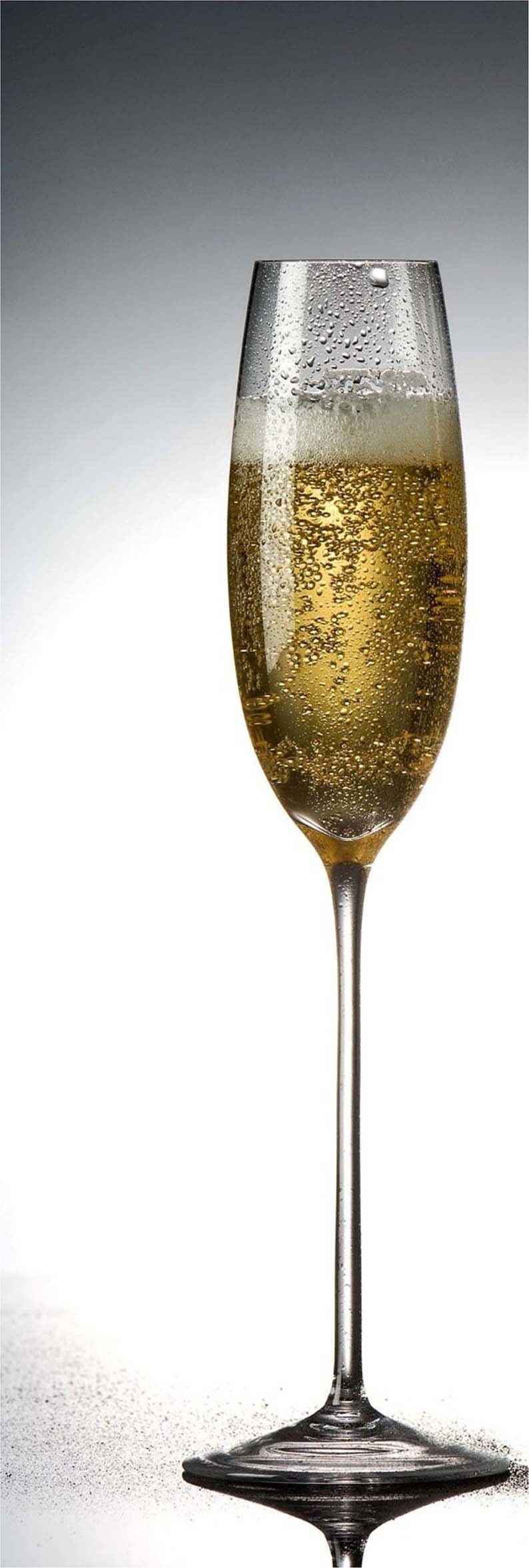
Champagne flute. Courtesy Fionn McCann

However, not all wine glasses are influenced by the Bauhaus. The champagne coupe is reputedly modelled on the breast of Marie Antoinette or Madame Pompadour (they must have been very small!) and more latterly Kate Moss and Claudia Schiffer have apparently allowed their breasts to be similarly used. My own preference is for the champagne flute (Fig. 5) definitely not modelled on a woman’s breast!

Who would have thought that the Bauhaus has influenced areas, which seem to be unrelated; hospital, wine glass, and label design. As Fiona MacCarthy says in her new book in a statement attributed to Walter Gropius: ‘If I have talent it is seeing the relationship of things’ [5].
